# Proteomic Analysis of Plasma-Derived Exosomes in Defining Their Role as Biomarkers of Disease Progression, Response to Therapy and Outcome

**DOI:** 10.3390/proteomes7030027

**Published:** 2019-06-30

**Authors:** Theresa L. Whiteside

**Affiliations:** Department of Pathology, University of Pittsburgh School of Medicine and UPMC Hillman Cancer Center, Pittsburgh, PA 15213, USA; whitesidetl@upmc.edu

Extracellular vesicles (EVs) have recently emerged as an intercellular communication system that plays an important role in health and becomes dysfunctional in disease. This system appears to be evolutionarily conserved, as all unicellular and multicellular organisms produce EVs [1]. In addition to carrying and delivering cellular messages, EVs are currently viewed as potential biomarkers of disease because their production levels and molecular content are altered with disease onset and progression [2]. EVs come in various sizes, have different cellular origins and carry distinct molecular and genetic cargos [3]. Larger EVs, such as apoptotic bodies, microvesicles (MVs) and oncosomes, are larger than 150 nm and are formed by apoptosis or are pinched-off the surface of parent cells, respectively. Exosomes are the only EVs that originate from the endocytic compartment, and because of this origin, their content includes components derived from the cell membrane (e.g., various integrins, cell adhesion molecules, immune or non-immune receptors and ligands) as well as from the endosomal membrane (e.g., proteins such as ALIX, TSG101 or syntenin). The cargo of exosomes reflects the molecular content of their parent cell and the sorting process used by this cell type [4]. Disease-related changes that might alter either the content or the exosome sorting and packaging processes are expected to be useful in diagnosis, estimates of disease activity or progression and of response to therapy. 

While exosomes are abundant in the circulation of healthy individuals, their concentration tends to increase with inflammation, cancer, autoimmune and infectious diseases [5]. Human tumors produce and release masses of exosomes, referred to as “tumor-derived exosomes” or TEX into body fluids. Tumor-derived exosomes (TEX) are being actively investigated as biomarkers that could serve as a tumor “liquid biopsy” which will faithfully recapitulate the molecular composition of parent tumor cells. Should this expectation materialize upon validation by comparisons of the exosome and the parent tumor contents, serial tumor liquid biopsies would become readily available using exosomes isolated from a small volume of the patient’s peripheral blood. In addition, TEX are emerging as potentially important surrogates of tumor-induced immune suppression in cancer and other diseases and as biomarkers of cancer responses to immune therapies [6]. Therefore, determinations of TEX molecular and genetic cargos are of great importance. 

Exosomes are the smallest of EVs (30–150 nm in diameter), but even this subset of EVs is heterogenous in size and the molecular content, because in body fluids, it represents a mix of exosomes derived from many different cell types [7]. In the tumor microenvironment (TME), they carry messages from the tumor to nonmalignant cells in the TME and in the periphery [7,8]. These messages are delivered to recipient cells as protein-mediated signals or as nucleic acids (e.g., mRNA/miRNA). They are delivered by exosomes first to the surface of recipient cells and then, upon exosome internalization, to the cytosol of recipient cells [8]. The result is reprogramming of the recipient cells that involves dramatic transcriptional changes of multiple genes that lead to alterations in the functional repertoire of these cells [9]. The TEX-reprogrammed nonmalignant cells in the TME, including mesenchymal stromal cells, immune cells, fibroblasts, endothelial cells, which now become a source of a new wave of exosomes that carry messages supporting tumor growth and its survival [9]. Thus, while TEX might serve as biomarkers of the tumor and its functional status, exosomes produced by reprogrammed nonmalignant cells in the TME could serve as biomarkers of the capability of the TME to promote tumor progression. 

The TEX-driven reprogramming described above suggests that molecular and genetic analyses of TEX as well as of exosomes produced by nonmalignant cells, the “non-TEX,” might be informative. However, exosomes present in plasma and other body fluids of cancer patients are mixtures of TEX and non-TEX. Isolation from body fluids of these exosome subsets can now be accomplished by immunoaffinity capture based on antibody-on-beads based separation of TEX and non-TEX [10]. The methodology involves isolation from precleared body fluids of total exosomes by size exclusion chromatography (SEC), separation of TEX from non-TEX by immune capture using Abs specific for antigens carried by TEX or for antigens carried by non-TEX, and finally detection and quantitation of the cargos carried by the separated exosome subsets [10]. Once fractionated, the detection and molecular profiling of the exosome protein cargo components can be accomplished by western blots (WB), on-beads flow cytometry and mass spectrometry [11]. 

At this time, a better understanding of the biological role of TEX and non-TEX and of mechanisms involved in reprogramming of the TME are necessary. While TEX serve as a an example, the same general approaches for isolation and characterization of exosomes from plasma or body fluids apply to diseases other than cancer. They also apply to exosomes in body fluids such as urine, saliva or ascites. Clearly, the potential significance of exosomes as biomarkers of disease progression, response to therapy and disease outcome requires further exploration. Proteomics are bound to become a critically important part of this exploration. However, while proteomic analysis of exosomes appears to be an attractive approach that has, in fact, been already broadly utilized, a number of challenges slowing down progress have materialized. Among them are the existing lack of EV‘s nomenclature, of uniformly defined criteria for exosome isolation from a mélange of various EVs and of established methodologies for EVs subtyping into functionally distinct subsets. Technical aspects of the proteome analysis in cancer to define TEX/non-TEX protein profiles characteristic for each tumor type are also a significant challenge. 

The presumption has been that exosomes from plasma of cancer patients would have a protein profile distinct from that of healthy donors and that a common exosome protein profile would characterize tumors with the same histology. Further, TEX protein profiles would be expected to be distinct from those of non-TEX. Almost immediately, this presumption proved to be difficult to test. Because exosome isolation and the nomenclature remain in flux, and there is no consensus in the field, samples submitted for mass spectrometry are often not exosomes (defined as 30–150 nm vesicles of the endocytic origin) but mixtures of small, intermediate or large vesicles with vastly different origins and characteristics. Numerous technical issues arose related to the prominent presence of “contaminating” plasma proteins, especially albumin, lipoproteins, and immunoglobulins, in plasma-derived exosomes as well as the need for sufficiently large exosome numbers and requirements for optimized protocols of sample processing prior to mass spectrometry [12]. As these technical issues were slowly solved, and detection of “true exosomal proteins” improved upon removal of the “contaminants,” a problem with sensitivity of protein detection emerged. Using WB or flow cytometry for detection of the exosome protein cargo, both methods being dependent on Ab-based amplification of specific proteins, it has been possible to measure and quantify biologically important proteins known to participate in functional reprogramming driven by TEX [11,13]. These proteins are not seen by untargeted mass spectrometry approaches for reasons that are likely to reflect their femtomolar quantities in exosomes. They are lost among large numbers (i.e., several hundred) of other proteins in the exosome cargo, and without Ab-based amplification, these proteins remain undetectable. The solution would be to increase exosome numbers provided for mass spectrometry to levels that may, however, be difficult to procure using currently available isolation methods. The paucity of Abs specific for antigens carried exclusively by tumor cells and TEX that can be used for separation of TEX from non-TEX is also a problem. Mixtures of Abs recognizing antigens highly overexpressed on tumor cells, such as EPCAM, EGFR or CSPG4, are useful for enrichment for TEX and have been used for the construction of microarrays for exosome capture from body fluids [14]. This strategy may yield sufficient material for protein quantification by flow cytometry but not for proteomics. Nevertheless, the availability of total exosomes that can be isolated from body fluids by existing methods and can be appropriately depleted of plasma-derived proteins in numbers sufficient for proteomic analysis may prove to be diagnostically and prognostically informative. 

The papers assembled in this volume illustrate various approaches and strategies developed to date for analysis of the EV proteomes in human diseases. The volume contains papers which range from reviews to methods to primary experimental papers—a cocktail of articles that should capture interest of individuals engaged in exosome research. The content includes e.g., a contribution by Fel et al. which compares proteomes of EVs isolated by ultracentrifugation from sera of patients with polycythemia vera and healthy donors; a paper by Smolarz et al. on purification of EVs by size exclusion chromatography from sera for proteomic profiling; and a paper by Hurvitz et al. on the role of integrins carried by EVs isolated from supernatants of breast cancer cell lines by precipitation and ultracentrifugation. These three papers, each utilizing a different EV isolation method, performed proteomics analyses that yielded interesting results, making it a difficult task to decide which method may be most advantageous in EV preparation for proteomics. At the other end of the spectrum, the volume includes three reviews by Surman et al., Abramowicz et al. and Jablonska et al., which consider the potential of proteomics analyses for discrimination of various clinical or physiological states in man. Overall, the enclosed contributions provide a view of the exosome proteomics as an emerging field that is still mostly at the developmental stage and may require future modifications and adaptations likely to be necessary when the universal criteria for EVs isolation and purification are established. Remarkably, given the existing challenges, as discussed above, exosome proteomics are yielding a wealth of provocative insights into the selective protein profiles of exosomes originating from various normal or malignant cells and suggest that exosome proteomics is here to stay and prosper in the future.
